# Intrahepatic Cholangiocarcinoma: A Summative Review of Biomarkers and Targeted Therapies

**DOI:** 10.3390/cancers13205169

**Published:** 2021-10-15

**Authors:** Alexandra W. Acher, Alessandro Paro, Ahmed Elfadaly, Diamantis Tsilimigras, Timothy M. Pawlik

**Affiliations:** The Ohio State University Wexner Medical Center, The James Comprehensive Cancer Center, Columbus, OH 43210, USA; Alexandra.Acher@hsc.utah.edu (A.W.A.); Alessandro.Paro@osumc.edu (A.P.); elfadaly.1@osu.edu (A.E.); Diamantis.Tsilimigras@osumc.edu (D.T.)

**Keywords:** intrahepatic, cholangiocarcinoma, biomarkers, outcomes, trials

## Abstract

**Simple Summary:**

Intrahepatic cholangiocarcinoma is the second most common primary liver malignancy. Among patients with operable disease, surgical resection is the cornerstone of therapy. Among the majority of patients who present with advanced disease treatment, systemic or targeted therapy is indicated. Recent advancements have provided more novel therapeutic approaches to a subset of patients with intrahepatic cholangiocarcinoma.

**Abstract:**

Although rare, intrahepatic cholangiocarcinoma (ICC) is the second most common primary hepatic malignancy and the incidence of ICC has increased 14% per year in recent decades. Treatment of ICC remains difficult as most people present with advanced disease not amenable to curative-intent surgical resection. Even among patients with operable disease, margin-negative surgical resection can be difficult to achieve and the incidence of recurrence remains high. As such, there has been considerable interest in systemic chemotherapy and targeted therapy for ICC. Over the last decade, the understanding of the molecular and genetic foundations of ICC has reshaped treatment approaches and strategies. Next-generation sequencing has revealed that most ICC tumors have at least one targetable mutation. These advancements have led to multiple clinical trials to examine the safety and efficacy of novel therapeutics that target tumor-specific molecular and genetic aberrations. While these advancements have demonstrated survival benefit in early phase clinical trials, continued investigation in randomized larger-scale trials is needed to further define the potential clinical impact of such therapy.

## 1. Introduction

Cholangiocarcinoma is defined by anatomic location and can arise throughout the biliary tree: distally, peri-hilar region, or intrahepatic. The different anatomic locations of cholangiocarcinoma correspond to varied etiologies of the disease. Specifically, extrahepatic cholangiocarcinoma likely derives from stem cells in peribiliary glands while intrahepatic cholangiocarcinoma (ICC) arises from hepatocyte stem cells [1,2]. In turn, the varied anatomic locations of cholangiocarcinoma can have implications for diagnosis, surgical planning, and resectability. In particular, ICC is the second most common primary hepatic malignancy. ICC is a rare cancer, with approximately 8000 cases diagnosed each year in the United States, comprising only 3% of gastrointestinal malignancies diagnosed globally per year [3,4]. The incidence of ICC has increased in recent decades, with some studies demonstrating a 14% increase in incidence per year since the early 1990s [5]. The increase in ICC incidence is likely due to the rising global prevalence of hepatitis C infection, as well as obesity associated non-alcoholic fatty liver disease and non-alcoholic steatohepatitis, which are known risk factors for ICC [5,6,7]. There are also regional differences in the risk factors associated with ICC. For example, the relative incidence of hepatolithiasis, liver fluke infection, as well as viral hepatitis is markedly higher among patients with ICC in Eastern countries. In contrast, among patients in Western countries, ICC is more often associated with primary sclerosing cholangitis and other diseases associated with chronic liver inflammation (non-alcoholic fatty liver disease, non-alcoholic steatohepatitis, and cirrhosis) [8].

Unfortunately, approximately one-half of patients who present with a new diagnosis of ICC will have advanced disease that is not amenable to curative-intent resection (R0 resection) [5]. While the best chance for cure, an R0 resection can only be achieved in 32–88% of patients who are candidates for curative-intent resection [5,9]. Unfortunately, even with R0 resection, 22% of patients will recur within 6 months of hepatectomy [10]. The majority (50%) of patients who recur have intrahepatic recurrence, while 20% have peritoneal recurrence and 20–30% recur with portal lymph node metastasis [11,12,13,14]. As such, long-term prognosis generally remains poor with a 5 year survival of 25% for patients with local disease, 8% for patients with regional disease, and 2% for patients with distant disease [4]. After controlling for patient and disease factors, patients who present with multiple tumors, tumor size (>5 cm), and lymph node metastases have a reduced 5 year overall survival [9,10,15]. Other machine-based learning platforms have also highlighted the importance of the cumulative impact of tumor size, number of tumors, clinical nodal status, and albumin–bilirubin grade on outcomes following curative-intent treatment of ICC [16].

Overall tumor response to systemic chemotherapy ranges from 10% to 30% [9,17,18]. While there are several randomized clinical trials examining the efficacy of adjuvant systemic therapies in ICC, a survival benefit has been most consistently demonstrated with gemcitabine plus cisplatin [19]. This was demonstrated in a randomized trial of gemcitabine plus cisplatin versus gemcitabine alone in patients with locally advanced or metastatic cholangiocarcinoma [20]. Median overall survival of patients with advanced or metastatic disease who received gemcitabine-cisplatin was 11.7 months versus 8.1 months for patients who received just gemcitabine (hazard ratio 0.64, 95% CI 0.52–0.8) [20]. Despite these results, 5 year survival with either therapy is poor [20]. As such, there has been increased interest in novel therapeutic targets to treat cholangiocarcinoma. 

Given the high incidence of advanced stage at presentation, poor prognosis associated with ICC, and the limited efficacy of current treatment modalities, there has been considerable interest in the molecular and genetic underpinnings of ICC [21]. Improved understanding of the molecular pathways that promote biliary oncogenesis and the genetic mutational foundations of ICC may elucidate potential targets for molecular and genetic-based treatment therapies, with the ultimate goal of improving overall survival for all-stage ICC. We herein review the pathogenesis of ICC and the associated molecular processes and genetic mutations that may promote biliary oncogenesis. We also describe novel and evolving targeted molecular and genetic-based therapies for the treatment of ICC. 

## 2. Pathogenesis of ICC

ICC most often occurs in the setting of chronic biliary inflammation and stasis. Patients with disease processes that promote chronic biliary stasis and inflammation (primary sclerosing cholangitis, hepatolithiasis, liver fluke infection, chronic hepatitis and cirrhosis) are therefore at risk of developing ICC [22,23]. Biliary stasis and inflammation promote excessive generation of nitric oxide, which potentiates oxidative damage to DNA and inhibits DNA repair and cellular apoptosis. Additionally, cyclo-oxygenase-2 (COX-2) is upregulated by induced nitric oxide species and acts to promote cholangiocyte growth and survival. Bile acids have also been demonstrated to facilitate cholangiocarcinogenesis through interference with cell signaling pathways and normal cellular apoptosis [22,23].

Common genetic mutations associated with ICC include KRAS, BRAF, TP53, and epidermal growth factor receptor [24,25,26,27,28,29,30]. KRAS mutations are the most commonly recognized mutation associated with ICC; however, their incidence varies widely from 8 to 53% [30]. BRAF mutations are also common and have a reported incidence of 0–22% in ICC tumors [30]. Interpretation of these data are complicated, however, as studies examining genetic mutations associated with cholangiocarcinoma typically categorize ICC with peri-hilar cholangiocarcinoma and distal cholangiocarcinoma, which may have different genetic mutational patterns [25,31,32]. In a study of isolated ICC, 7% of ICC tumors were associated with KRAS mutations and 7% had BRAF mutations [30]. In this study, tumors associated with KRAS and BRAF mutations also had a more advanced TNM stage mostly due to lymph node metastasis. Tumor size, number, grade, evidence of vascular invasion or perineural invasion, or satellite lesions did not correlate with mutational status. Patients with tumors exhibiting either KRAS or BRAF mutations had worse median survival versus wild-type tumors (23 months versus 34 months, respectively, *p* = 0.05) [30]. Patients with KRAS mutations also had a worse 5 year overall survival compared with patients who had ICC tumors with BRAF mutations (13.5 months vs. 23.2 months, respectively, *p* = 0.05) [30].

**Figure 1:**
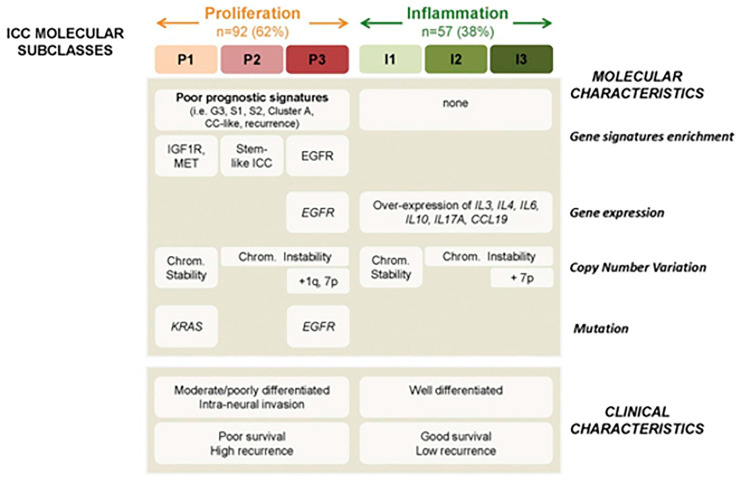
ICC Molecular Subclasses, reproduced from Sia et al. [32]. Summary of characteristics of ICC classes. Specific molecular and clinical characteristics differ between ICC classes. Molecular characteristics such as signatures of poor prognosis (i.e., cluster A, CC-like, G3, S1, S2, and stem-cell like ICC), oncogenic pathways (i.e., IGF1R, MET, EGFR), gene expression (i.e., *EGFR*, *IL*s), copy number variations, and oncogenes mutations (*KRAS* and *EGFR*) are differentially enriched in the proliferation and inflammation classes. Clinical characteristics such as moderate/poorly differentiated tumors and intraneural invasion are more frequent in the proliferation class. Differences in survival and recurrence were observed.

More recently, studies employing gene expression profiling, high-density single-nucleotide array, and mutation analyses of formalin-fixed ICC samples have identified two predominant biological types of ICC: inflammatory and proliferative (Figure 1) [32]. These analyses integrate components of previously distinct hypotheses on the epigenetic, molecular, and genetic origins of ICC. Inflammatory type ICC accounts for 38% of ICC and is defined by activation of pro-inflammatory signaling pathways via interleukin-10 (IL-10), interleukin-4 (IL-4), interleukin-6 (IL-6), overexpression of other cytokines, and activation of signal transducer and activator of transcription 3 protein (STAT3) (Figure 1) [32]. STAT3 has been noted to play a role in many types of cancer and has been associated with a worse prognosis in specific cancers. STAT3 is activated by upstream cytokines and growth factors, including IL-6 and human epidermal growth factor (EGFR) [33]. STAT3 relays signals from activated cytokine and growth factor-receptors at the cellular plasma membranes to the nucleus to promote gene transcription that promotes cellular differentiation, proliferation, angiogenesis, and immune response and inhibits apoptosis [33]. Gene expression that is induced by STAT3 produces cytokines that can re-activate STAT3 (i.e., IL-6), leading to an intrinsic and cyclic propagation of this oncogenic pathway [34]. Therefore, in conditions of chronic biliary inflammation and stasis that activate STAT3, STAT3 also acts to propagate local inflammation and oncogenesis. 

The proliferative type of ICC accounts for 62% of ICC and is defined by activation of oncogenic signaling pathways, most notably through receptor tyrosine kinases (RTK) [32]. RTKs are cellular plasma membrane receptor proteins (consisting of an extracellular ligand binding domain, transmembrane helix, and intracellular domain) that mediate cellular communication and signaling [35]. There are 58 RTKs that are encoded within the human genome, which are grouped into 20 different classes, differentiated by signaling pathway and function [35]. The extracellular domain of RTKs binds growth factor ligands to signal downstream intracellular processes, including cellular proliferation, differentiation, motility, and metabolism [36]. Aberrant expression and mutations within genes encoding the component parts of RTKs (i.e., the extracellular domain, transmembrane helix, or the intracellular domain) promote various mechanisms of abnormal RTK function. The main mutations types that drive RTK dysfunction are gain of function mutations (EGFR), overexpression and genomic amplification of RTKs (EGFR, MET), chromosomal rearrangements (fusion of genes encoding BCR and ABL tyrosine kinases, or PDGFR tyrosine kinases), and constitutive activation by kinase domain duplication [36]. Furthermore, in subgroup analyses, Sia et al. demonstrated that proliferative-type ICC could be further divided into subclasses (named P1–P3), with distinct prognostic implications reflective of their gene expression predominance [32]. Proliferative-type ICC also demonstrated enrichment of several oncogenic pathways, that are described in hepatocellular cancer (cluster A, G3 proliferation, S1 signature), which may help explain its relatively worse prognosis when compared with inflammatory type ICC [32].

When comparing clinical phenotypes of inflammatory versus proliferative ICC, several important differences have been described. For example, proliferative-type ICC tumors are more likely to be moderately to poorly differentiated, while inflammatory type tumors are more likely to be well differentiated. Survival analyses of proliferative versus inflammatory type ICC reveal that patients with proliferative-type ICC have shorter time to recurrence (15 vs. 37 months, *p* = 0.03) and a reduced median survival (24.3 vs. 47.2, *p* = 0.048) [32]. In addition, the incidence of KRAS mutations was 8% in proliferative-type ICC tumors and 7% in inflammatory type ICC tumors; the incidence of BRAF mutations was 5% among proliferative-type ICC tumors and 2% among inflammatory type ICC tumors [32]. The proliferative class of ICC may share common progenitor cells with HCC, which may explain the similar aggressive phenotypes. On sub-analyses that utilized DNA copy number variation and gene expression-based classes to predict recurrence and survival, certain subclasses of the proliferative-type ICC had poor prognostic signatures similar to signatures associated with HCC (Figure 1) [32]. This hypothesis may also be supported by the observation that progenitor cells, which give rise to both hepatocytes and cholangiocytes, are activated in adult human livers by hepatocyte replicative senescence and microenvironment inflammation and damage. Therefore, in the right epigenetic context, progenitor cells may give rise to both HCC and ICC or even cases of combined HCC-ICC [37,38]. 

The tumor microenvironment has also been used to define genomic and molecular subgroupings related to cholangiocarcinoma. To this point, Job et al. described four subtypes of ICC that were distinguished by cell type and functional transcriptomic markers within tumor specific microenvironments [39]. These subtypes correlated with prognosis and overall survival, highlighting that immunogenic ICC subtypes may be more amenable to treatment with immune checkpoint inhibitors than others ICC lesions [39]. Similarly, Andersen et al. profiled transcriptomes from resected cholangiocarcinoma within the context of their tumor microenvironment and identified certain microenvironment characteristics associated with worse prognosis [40]. These investigators also reported better treatment response to lapatinib, a dual inhibitor of EGFR and HER2, as well as trastuzumab, a selective inhibitor of HER2, among specific ICC subtypes [40].

Despite the considerable progress made in understanding the molecular and genetic underpinnings of ICC in recent decades, many of these studies have been only hypothesis generating. The data do, however, suggest a complicated network of microenvironment, molecular, and genetic factors that strongly influence cholangiocarcinogenesis. Importantly, there may be several different ICC phenotypes that reflect differences in etiology and pathogenesis. In turn, better characterization of different phenotypic-genomic ICC tumors has facilitated the investigation of novel and targeted treatment approaches for ICC. 

## 3. Identified Targetable Mutations

**Figure 2:**
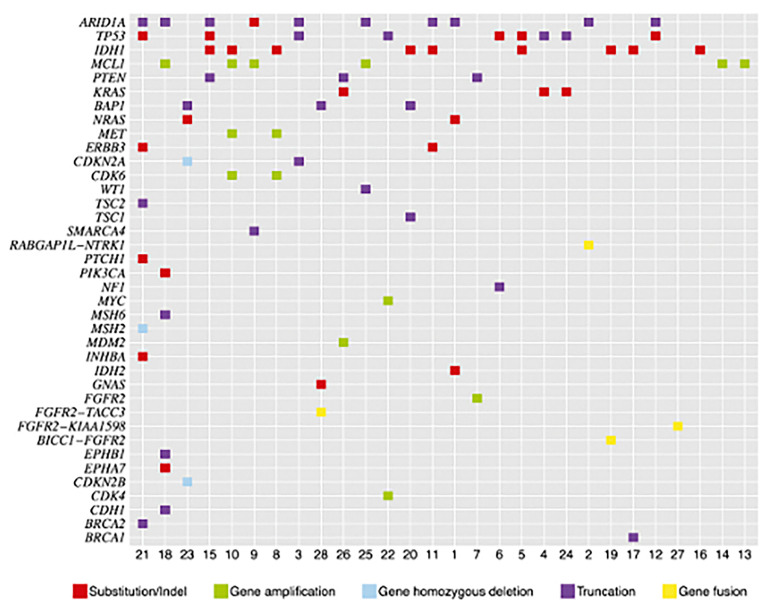
Tile plot of genomic alterations in 28 cases of ICC, reproduced from Ross et al. [41].

The development of next-generation DNA sequencing has shifted the treatment landscape for many different types of cancer including ICC. In fact, up to 70% of ICC tumors may have at least one targetable gene aberration and on average have anywhere from 1 to 2 targetable mutations per tumor examined [41,42]. Several early small studies observed the most common mutations in ICC within AT-rich interactive domain-containing protein 1A (ARID1A, 36%), isocitrate dehydrogenase 1/2 (IDH1/2, 36%), TP53 (36%), and Myeloid Leukemia and Chlamydia (MCL1, 21%) genes [41]. Common actionable mutations (those with FDA-approved drugs available for treatment) included fibroblast growth factor receptor 2 (FGFR2, 14%), KRAS (11%), phosphatase and tensin homolog (PTEN, 11%), cyclin-dependent kinase inhibitor 2A/B (CDKN2B, 7%), Erb-B2 Receptor Tyrosine Kinase 3 (*ERBB3*, 7%), *MET* (7%), *NRAS* (7%), *CDK6* (7%), *BRCA1* (4%), *BRCA2 (4%), NF1* (4%), *PIK3CA* (4%), *PTCH1* (4%), and *TSC1* (4%) genes (Figure 2) [41]. More recently, other larger studies have demonstrated that the most frequent genetic alterations in ICC occur to be in TP53 (TP53; 27%), cyclin-dependent kinase inhibitor 2A/B (CDKN2A/B; 27%), KRAS (22%), AT-rich interactive domain-containing protein 1A (ARID1A; 18%), and isocitrate dehydrogenase 1/2 (IDH1; 20%), BRCA1 associated protein (BAP1, 15%), FGFR2 (11%), and MET (2%) genes [42]. After controlling for patient and disease factors, multivariable analyses demonstrate that TP53 aberrations were associated with worse survival (HR 1.68, *p* = 0.015), while FGFR aberrations were associated with improved overall survival (HR 0.478, *p* = 0.03) [42]. 

Programmed Death-Ligand 1 (PD-L1) expression has also been reported to be present in 10–70% of ICC tumor specimens [43,44,45]. PD-L1 positivity has been associated with more aggressive ICC characteristics and worse survival [43]. While uncommon, microsatellite-instability (MSI) has been explored as a biomarker and target for personalized ICC therapy. Due to the rarity of MSI in ICC, definitive conclusions regarding its incidence and prognostic implications have been difficult to decipher. The available data suggest that microsatellite unstable tumors (as defined by loss of DNA mismatch repair proteins MLH1, PMS2, MSH2, MSH6) are uncommon and occur only in a minority of patients with ICC (ranges from 1 to 10%) [43,46]. 

## 4. Results of Targeted Therapies for ICC

**Figure 3:**
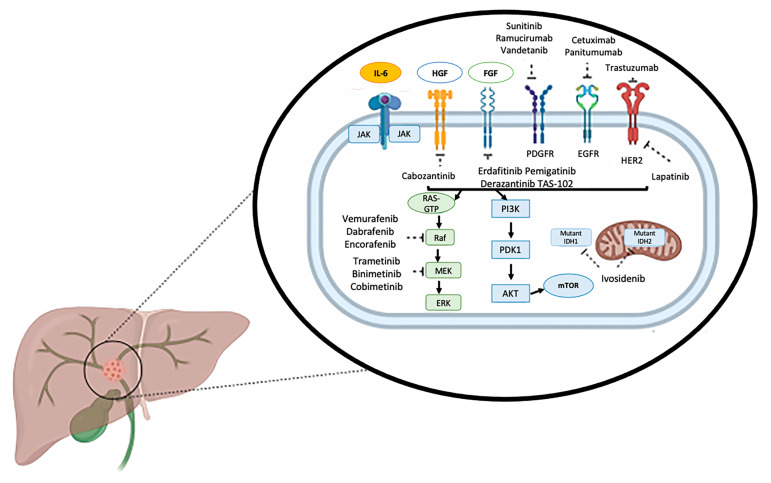
Schematic representation of therapeutically relevant signaling pathways and selected targeted therapies currently under evaluation in biliary tract cancer, reproduced from Rizzo et al. [47]. AKT: protein kinase B; EGFR: epidermal growth factor receptor; FGF: fibroblast growth factor; HER2: epidermal growth factor receptor 2; HGF: hepatocyte growth factor; IL-6: interleukin 6; IDH: isocitrate dehydrogenase; JAK: Janus kinase; mTOR: mammalian target of rapamycin; PDGFR: platelet derived growth factor receptor; PDK1: phosphoinositide-dependent kinase-1; PI3K: phosphoinositide 3-kinase.

The evolution in the understanding of molecular and genetic pathways associated with ICC has facilitated several early phase clinical trials. These studies have generally investigated novel targeted therapies used either as mono-therapy or in combination with other systemic therapies. Novel therapies and their genetic or molecular targets has been well summarized in schematic form by Rizzo et al. [43] (Figure 3). Although various genetic aberrations have been identified in ICC, the incidence of many mutations remain low, which has been made the use of targeted therapy challenging. Early phase clinical trials have been initiated, however, for some of the more commonly occurring genetic aberrations including those involving IDH1/2, FGFR, EGFR, and VEGF [21,43]. 

### 4.1. Targeted Therapy: Isocitrate Dehydrogenase

Since IDH1/2 mutations are present in approximately 10–20% of ICC lesions, this genetic aberration has been the target of therapeutic intervention [41]. The mechanism by which IDH aberrations promote cholangiocarcinogenesis are still being fully elucidated. Animal models suggest that mutation of IDH induces an abnormal response to hepatocyte injury and inflammation, as well as silencing of HNF4-alpha, a transcription factor crucial to hepatocyte differentiation that is a potent anti-proliferative and tumor suppressor. In mouse models, mutant IDH-associated silencing of HNF4-alpha has resulted in a pro-neoplastic state with a biliary predominance [48]. 

The safety and efficacy of Ivosidenib, an inhibitor of mutated IDH1, have been investigated in a multicenter randomized double-blinded placebo-controlled phase 3 trial among patients with advanced cholangiocarcinoma who had progressed on either fluorouracil or gemcitabine-based chemotherapy [49]. The trial included patients with all types cholangiocarcinoma (i.e., intrahepatic, hilar, distal); however, the majority of accrued patients had ICC (89% in treatment arm and 77% in placebo arm) [49]. Among the 185 patients (124 in treatment arm and 61 in placebo arm), Ivosidenib was associated with an improved progression-free survival (median 2.7 months vs. 1.4 months, HR 0.37, 95% CI 0.25–0.54, *p* < 0.0001) and comparable safety profiles (30% of patients in the treatment arm patients had serious adverse events vs. 22% in the control arm) [49]. While other phase 1 and 2 studies have examined the safety profiles of inhibitors of mutated IDH1/2, a definitive benefit in the treatment of ICC has yet to be established [50].

### 4.2. Targeted Therapy: Fibroblast Growth Factor Receptor

FGFR aberrations have been identified in 10–15% of ICC tumors [41,42,51]. FGFR is expressed on multiple cell types and consists of four transmembrane receptors (FGFR1–4) with intracellular tyrosine kinase domains. The binding of these FGFR receptors leads to unregulated activation of several cellular proliferation pathways, including RAS-Raf-MAPK, JAK-STAT, and PI3-AKT-mTOR, leading to angiogenesis and unregulated cellular proliferation [52]. 

Pemigatinib is an oral kinase inhibitor, which inhibits FGFR 1, FGFR2, and FGFR3. Several Phase 2 trials have examined the safety and antitumor activity of Pemigatinib among patients with advanced cholangiocarcinoma with FGFR rearrangement. In one trial, among 146 enrolled patients, 35% had an objective treatment response, 42% of patients died from disease progression, and 45% of patients had serious adverse events [53]. Pemigatinib is currently being investigated in a phase 3, open-label, randomized, active-controlled multicenter trial that compares efficacy and safety of Pemigatinib versus standard of care (gemcitabine-cisplatin) in patients with advanced or metastatic cholangiocarcinoma with FGFR2 aberration (ClinicalTrials.gov, accessed on 21 September 2021, Identifier: NCT03656536). Futibatinib is a different highly selective irreversible oral inhibitor of FGFR 1–4. Its safety has been studied in a multicenter, phase 2 trial of patients with advanced or metastatic ICC with FGFR2 gene rearrangements who had disease progression after first line therapy (gemcitabine-cisplatin). Among 103 patients who enrolled, 34% had an objective response and the disease control rate was 76% at ≥6 months follow-up. Serious adverse events were experienced by 73% of patients (≥grade 3) [52]. The safety and efficacy of Futibatinib are now being compared with standard of care (gemcitabine-cisplatin) in a multicenter phase 3 study of patients with advanced cholangiocarcinoma with *FGFR2* gene rearrangements (NCT04093362). In addition to these more well-known drugs, there are a number of other early phase studies examining drug safety and efficacy of inhibitors of mutant FGFR, as well as several upstream or downstream processes related to FGFR-based pathways [42,50]. Javle et al. published results from a single-arm phase II study that examined efficacy and safety of infigratinib, a FGFR-selective tyrosine kinase inhibitor, in patients with previously treated advanced cholangiocarcinoma [49,51,54]. In a cohort of 108 patients with advanced or metastatic cholangiocarcinoma, the objective response rate was 23%, the median duration of response was 5.0 months, and the median progression-free survival was 7.3 months (95% CI 5.6–7.6 months) [51,54,55]. The majority of patients experienced hyperphosphatemia despite taking a prophylactic phosphate binder (77%) and non-severe eye disorders (68%), while a minority of patients experienced serious eye disorders (16%, central serous retinopathy and retinal pigment epithelium detachment) [54,55]. Based on these results, a phase III clinical trial of infigratinib versus standard of care gemcitabine/cisplatin is currently being conducted (NCT 03773302). Additional ongoing trials examining treatment efficacy and safety of FGFR targeted therapies are listed in Table 1.

### 4.3. Targeted Therapy: Epidermal Growth Factor Receptor

The HER tyrosine kinase family includes EGFR and HER1–4 aberrations. EGFR aberrations have been identified in 25% of ICC tumors [56]. EGFR is a subclass of the tyrosine kinase transmembrane receptor that binds to epidermal growth factor and activates signaling pathways involved in cell motility, cell adhesion, angiogenesis and invasion [57]. HER/EGFR aberration causes unregulated activation of these pathways.

Several early phase trials examining safety and efficacy of inhibitors of aberrant EGFR and HER (lapatinib, erlotinib, pertuzumab, trastuzumab) in patients with cholangiocarcinoma and other solid tumors are either pending or have failed to show significant objective treatment response. Analyses, have been limited, however, by patient selection and heterogeneity of diagnosis [50,58].

### 4.4. Targeted Therapy: Immune Checkpoint Inhibitors

Programmed Death-Ligand 1 (PD-L1) has been noted in 10–70% of ICC tumor specimens and its expression has been associated with tumor aggressiveness and diminished survival [43]. PD-L1 is expressed on tumor cells and binds PD-1 receptors on activated T cells to inhibit cytotoxic action, thereby resisting the immune mediated defense. The safety and efficacy of a number of PD-L1 inhibitors have been examined in relation to advanced or metastatic PD-L1-positive cholangiocarcinoma (Table 2) [43]. These early phase trials failed to demonstrate definitive drug related benefits, yet initial results do suggest potential for both treatment efficacy and safety [43]. As such, while MSI-ICC is rare and small study numbers preclude robust analyses, some studies have reported relatively prolonged survival of patients with MSI-ICC treated with targeted immune checkpoint inhibitors. There are a number of ongoing early phase trials evaluating immune checkpoint inhibitor treatment efficacy and safety in patients with advanced MS- ICC (Table 3) [43].

### 4.5. Targeted Therapies: BRAF Mutations

BRAF mutations occur in 5–7% of ICC [63]. BRAF is a tyrosine kinase member of the RAS-RAF-MEK-ERK pathway (mitogen-activated protein kinase, MAPK), which is an integral pathway that mitigates cell proliferation, differentiation, transformation, and apoptosis [64]. BRAF mutations have been associated with higher TNM stage, resistance to systemic chemotherapies, and worse survival [26,30]. Targeted therapies pertaining to BRAF-positive ICC have demonstrated mixed results [65]. Many of studies to date have been “bucket” studies that included many different kinds of solid tumors harboring BRAF-mutations [65]. As such, interpretation of these data and the identification of disease-specific efficacy has been challenging. While several ongoing trials pertaining to patients with metastatic biliary tract cancer are ongoing, results from these trials are still pending (Table 4) [65].

## 5. Conclusions

Considerable progress has been made in understanding the molecular and genetic pathogenesis of ICC in recent decades. This evolution in knowledge has facilitated development and study of novel targeted therapies for patients with advanced ICC. While some targeted therapies have demonstrated significant potential relative to disease progression and even survival, the data are still emerging. Notwithstanding these limitations, patients with advanced ICC should have genetic profiling performed to identify potential targeted therapies. As with many rare disease, it is critical that patients be appropriately enrolled in clinical trials to help better define the role, efficacy and safety of targeted therapies for ICC. 

## Figures and Tables

**Table 1:** Ongoing trials evaluating FGFR and IDH1/2 inhibitors among patients with advanced solid tumors or ICC with or without known FGFR or IDH1/2 mutations (clinicaltrials.gov, accessed on 21 September 2021).

NCT No.	Phase	Setting	Arm A	Arm B	Agent Description	Primary Outcomes
01752920	I/II	Advanced solid tumor with FGFR alteration	Derazantinib		FGFR inhibitor	NAE
02150967	II	Advanced cholangiocarcinoma, FGFR2 gene mutation	BGJ398		BGJ398: FGFR inhibitor	ORR
02465060		Advanced solid tumors, lymphomas or multiple myeloma	Multiple genetic-alteration-specific drugs		Diverse mechanisms	ORR
02924396	II	Advanced cholangiocarcinoma	Pemigatinib		FGFR2 inhibitor	ORR
03230318	II	ICC or HCC with FGFR gene fusions	Derazantinib		FGFR inhibitor	ORR, PFS at 3 months
03656536	II	Advanced bile duct cancer	Pemigatinib	Gemcitabine + cisplatin	Pemigatinib: FGFR inhibitor	PFS
03773302	III	Advanced cholangiocarcinoma with FGFR2 gene mutation	BGJ398	Gemcitabine + cisplatin	BGJ398: FGFR inhibitor	PFS
04093362	III	Advanced cholangiocarcinoma with FGFR2 rearrangements	TAS-120/Futibatinib	Gemcitabine + cisplatin	TAS-120: FGFR inhibitor	PFS
0421168	II	Advanced biliary tract cancer	Toripalimab + Lenvatinib		Toripalimab: Recombinant anti-human PDI IgG4Lenvatinib: angiogenesis inhibitor that targets multiple tyrosine kinases including FGFR	ORR
04238715	II	Advanced cholangiocarcinoma with FGFR2 gene fusion	E7090		FGFR2 inhibitor	ORR
04256980	II	Advanced cholangiocarcinoma including those with FGFR2 rearrangements	Pemigatinib		FGFR2 inhibitor	ORR
04353375	II	Advanced ICC with FGFR2 fusion	HMPL-453		FGFR inhibitor	ORR at 6 months
04526106	I	Advanced solid tumor with FGFR2 amplification, mutation, rearrangement, translocation, activation	RLY-4008		FGFR2 inhibitor	MTD, NAE
04919642	II	Advanced cholangiocarcinoma with FGFR mutation	TT-00420		Multi-kinase inhibitor including FGFR	ORR
05039892	II	Advanced bile duct cancer with FGFR2 mutation	3D185		FGFR inhibitor	ORR
03212274	II/III	Advanced solid neoplasms with IDH1/2 mutation	Olaparib		PARP inhibitor	ORR
04521686	II/III	Advanced solid neoplasms with IDH1/2 mutation	LY3410738		mIDH1 inhibitor	ORR
03878095	II/III	Advanced solid neoplasm with IDH1/2 mutation	Olaparib + Ceralasertib		ATR inhibitor	ORR

Ongoing trials evaluating FGFR and IDH1/2 inhibitors in patients with advanced solid tumors or ICC with or without known FGFR or IDH1/2 mutations, as listed on clinicaltrials.gov, accessed on 21 September 2021. HCC, hepatocellular carcinoma; ICC, intrahepatic cholangiocarcinoma; FGFR, fibroblast growth factor receptor; MTD, maximum tolerated dose; NAE number adverse events; ORR objective response rate; PFS, progression-free survival; PD1, programmed cell death protein.

**Table 2:** Ongoing Phase I-III clinical trials evaluating immune checkpoint inhibitors in biliary tract cancer patients with advanced disease [43].

NCT No.	Phase	Setting	Arm A	Arm B	Agent Description	Primary Outcomes
04066491	II/III	First line	Bintrafusp alfa (M7824) plus CisGem	Placebo + CisGem	Bintrafusp alfa: bifunctional fusion protein composed by PD-L1 antibody fused with 2 extracellular domains of TGF-B receptor	DLTs, OS
03875235	III	First line	Durvalumab + CisGem	Placebo + CisGem	Durvalumab: PD-L1 inhibitor	OS
03260712	II	First line	Pembrolizumab + CisGem		Pembrolizumab: PD-L1 antibody	PFS at 6 months
04300959	II	First line	Anlotinib + tremelimumab + CisGem	CisGem	Anlotinib: TKI inhibiting PDGFR, FGFR, VEGFR, c-KIT kinase Sintilimab: PD-L1 inhibitor	OS at 12 months
03046862	II	First line	Durvalumab + tremelimumab + CisGem		Durvalumab: PD-L1 inhibitor Tremelimumab: anti-CTLA-4 agent	
03796429	II	First line	Toripalimab + S-1 + Gem		Toripalimab: PD-1 antibody	PFS, OS
04172402	II	First line	Nivolumab + S-1 + Gem		Nivolumab: PD-1 antibody	ORR
04027764	II	First line	Toripalimab + S-1 + albumin + paclitaxel		Toripalimab: PD-1 antibody	ORR
03478488	III	First line	KN035 + GEMOX	GEMOX	KN035: PD-L1 inhibitor	OS
04191343	II	First line	Toripalimab + GEMOX		Toripalimab: PD-1 antibody	ORR
04003636	III	First line	Pembrolizumab + CisGem	Placebo + CisGem	Pembrolizumab: PD-L1 antibody	PFS, OS
03937895	I/II	First or later line	Pembrolizumab + allogenic NK cell (SMT-NK)		Pembrolizumab: PD-L1 antibody SMT-NK: allogenic natural killer cell	DLTs, ORR
03639935	II	Maintenance after platinum-based first line	Nivolumab + Rucaparib		Nivolumab: PD-1 antibody Rucaparib: PARP inhibitor	PFS at 4 months
03785873	I/II	Second line	Nivolumab + 5-FU + Nallri		Nivolumab: PD-1 antibody	DLTs, PFS
04298021	II	Second line	AZD6738 + Durvalumab	AZD6738 + Olaparib	AZD6738: ATR and ATM inhibitor Durvalumab: PD-L1 inhibitor Olaparib: PARP inhibitor	DCR
03110328	II	Second line	Pembrolizumab		Pembrolizumab: PD-L1 antibody	PFS, OS
04211168	II	Second line	Toripalimab + Lenvatinib		Toripalimab: PD-L antibody Lenvatinib: TKI	ORR, AEs
03797326	II	Second line	Pembrolizumab + Lenvatinib		Pembrolizumab: PD-L1 antibody Lenvatinib: TKI	
04010071	II	Second line	Toripalimab + axitinib		Toripalimab: PD-1 antibody Axitinib: TKI	ORR, PFS
03704480	II	Second line	Durvalumab + tremelimumab	Durvalumab + tremelimumab + paclitaxel	Durvalumab: PD-L1 inhibitor Tremelimumab: anti-CTLA-4 agent	PFS
04003636	III	First line	Pembrolizumab + CisGem	Placebo + CisGem	Pembrolizumab: PD-1 antibody	PFS, OS
03937895	I/II	First or later line	Pembrolizumab + allogenic NK cell (SMT-NK)		Pembrolizumab: PD-1 antibody SMT-NK: allogenic natural killer	DLTs, ORR
03999658	II	Second or later line	STI-3031		St3031: PD-L1 inhibitor	ORR
03475953	I/II	Second or later line	Avelumab + regorafenib		Avelumab: PD-L1 inhibitor Regorafenib: TKI	RP2D
03801083	II	Second or later line	TILs		TILs: tumor-infiltrating Lymphocytes	ORR
04057365	II	Second or later line	Nivolumab + DKN-01		Nivolumab: PD-1 antibody DKN-01: humanized monoclonal antibody against DKK1 protein	ORR
04298008	II	Third line	AZD6738 + Durvalumab		AZD6738: ATR and ATM inhibitor Durvalumab: PD-L1 inhibitor	DCR

Ongoing phase I to III clinical trials evaluating immune checkpoint inhibitors in biliary tract cancer patients with advanced disease, reproduced from Rizzo et al. [43]. This table includes ongoing clinical trials assessing immunotherapy as first-, second-, or later-line treatment. 5-FU: 5-fluorouracil; AEs, adverse events; ATM, ataxia-telangiectasia mutation; BTC, biliary tract cancer; CisGem, cisplatin plus gemcitabine combination; CTLA-4, cytotoxic T-lymphocyte antigen 4; DCR: disease control rate; DLTs, dose-limiting toxicities; FGFR, fibroblast growth factor receptor; GBC, gallbladder cancer; GEMOX, gemcitabine plus oxaliplatin; ORR, overall response rate; OS, overall survival; PARP, poly ADP ribose polymerase; PDGFR, platelet-derived growth factor receptor; PD-1, programmed death 1, PFS, progression-free survival; RP2D, recommended phase II dose; S-1: tegafur/gimeracil/oteracil; TILs: tumor-infiltrating lymphocytes; TKI, tyrosine kinase inhibitor; VEGFR, vascular endothelial growth factor.

**Table 3:** Reported outcomes of single-agent immune checkpoint inhibitors in advanced biliary tract cancer (BTC) [43].

Phase	Setting	Immune Check Point Inhibitor	Agent Description	Outcomes
Ib [58]	Second line or later	Pembrolizumab	Pembrolizumab: PD-1 inhibitor	mPFS 1.8 months mOS 5.7 months ORR 13% SD rate 17%
II [58]	Second line or later	Pembrolizumab	Pembrolizumab: PD-1 inhibitor	mPFS 2.0 months mOS 7.4 months ORR 5.8%
II [59]	Second line or later	Nivolumab	Nivolumab: PD-1 inhibitor	mPFS 1.4 months mOS 5.2 months PR rate 3%
II [60]	Second line or later	Nivolumab	Nivolumab: PD-1 inhibitor	mPFS 3.7 months mOS 14.2 months ORR 20% DCR 50%
II [61]	Second line or later	Durvalumab	Durvalumab: PD-L1 inhibitor	mPFS 1.5 months mOS 8.1 months PR rate 4.2%
I [62]	Second line or later	M7824	M7824: PD-L1 inhibitor	mOS 12.7 months ORR 02%

Reported outcomes of single-agent immune checkpoint inhibitors in advanced biliary tract cancer (BTC), reproduced from Rizzo et al. [43].

**Table 4:** Ongoing trials evaluating BRAF targeted therapies in advanced biliary tract cancer registered on ClinicalTrials.gov, accessed on 21 September 2021 [65].

NCT No.	Phase	Setting	Arm A	Arm B	Agent Description	Primary Outcomes
04190328	I	Second or later line; BRAF-mutant solid tumors, including BTC	ABM-1310		ABM-1310: BRAF inhibitor	MTD/RP2D
04249843	I	Second or later line; BRAF-mutant solid tumors, including BTC	BGB-3245		BGB-3245: BRAF inhibitor	DLT MTD/RP2D
03839342	II	Second or later line; BRAF-mutant solid tumors, including BTC	Binimetinib + encorafenib		Binimetinib: BRAF inhibitor Encorafenib: BRAF inhibitor	ORR
01989585	I/II	Second or later line; BRAF-mutant solid tumors, including BTC	Dabrafenib + trametinib	Dabrafenib + trametinib + navitoclax	Dabrafenib: BRAF inhibitor Trametinib: BRAF inhibitor Navitoclax: Bcl-2 inhibitor	MTD CR rate
04418167	I	Second or later line; BRAF-mutant solid tumors, including BTC with MAPK pathway mutations	JSI-I 187	JSI-I 187 + dabrafenib	JSI-I 187: ERK inhibitor Dabrafenib: BRAF inhibitor	AEs
03272464	I	Second or later line; BRAF-mutant solid tumors, including BTC	Dabrefenib + trametinib + itacitinib		Dabrafenib: BRAF inhibitor Trametinib: BRAF inhibitor Icatinib: JAK I inhibitor	MTD

Ongoing trials evaluating BRAF-targeted therapies in advanced biliary tract cancer, reproduced from Rizzo et al. [65]. Abbreviations: AEs: adverse events; BTC: biliary tract cancer; CR: complete response; DLTs: dose-limiting toxicities; MTD: maximum tolerated dose; JAK1: Janus-associated kinase 1; MEK: mitogen-activated protein kinase; ORR: overall response rate; RP2D: recommended phase 2 dose.
